# The effect of scan parameters on T1, T2 relaxation times measured with multi-dynamic multi-echo sequence: a phantom study

**DOI:** 10.1007/s13246-022-01128-0

**Published:** 2022-05-13

**Authors:** Zuofeng Zheng, Jiafei Yang, Dongpo Zhang, Jun Ma, Hongxia Yin, Yawen Liu, Zhenchang Wang

**Affiliations:** 1 Department of Radiology, Beijing Friendship Hospital, Capital Medical University, Yong An Road 95, Beijing, 100050 China; 2 Department of Radiology, Beijing ChuiYangLiu Hospital, Beijing, China; 3 School of Biological Science and Medical Engineering, Beihang University, Beijing, China

**Keywords:** Synthetic magnetic resonance imaging, Scan parameter, T1 relaxation, T2 relaxation

## Abstract

Multi-Dynamic Multi-Echo (MDME) Sequence is a new method which can acquire various contrast-weighted images using quantitative relaxometric parameters measured from multicontrast images. The purpose of our study was to investigate the effect of scan parameters of MDME Sequence on measured T1, T2 values of phantoms at 3.0 T MRI scanner. Gray matter, white matter and cerebrospinal fluid simulation phantoms with different relaxation times (named GM, WM, CSF, respectively) were used in our study. All the phantoms were scanned 9 times on different days using MDME sequence with variations of echo train length, matrix, and acceleration factor. The T1, T2 measurements were acquired after each acquisition. The repeatability was characterized as the intragroup coefficient of variation (CV) of measured values over 9 times, and the discrepancies of measurements across different groups were characterized as intergroup CVs. The highest intragroup CVs of T1-GM, T2-GM, T1-WM, T2-WM, T1-CSF, T2-SCF were 1.36%, 1.75%, 0.74%, 1.41%, 1.70%, 7.79%, respectively. The highest intergroup CVs of T1-GM, T2-GM, T1-WM, T2-WM, T1-CSF, T2-SCF were 0.54%, 1.86%, 1.70%, 0.94%, 1.00%, 2.17%, respectively. Quantitative T1, T2 measurements of gray matter, white matter and cerebrospinal fluid simulation phantoms derived from the MDME sequence were not obviously affected by variations of scanning parameters, such as echo train length, matrix, and acceleration factor on 3T scanner.

## Introduction

Magnetic resonance imaging (MRI) is widely used in clinical practice for evaluating pathologies because of its excellent soft tissue contrast. Quantitative MRI has been gaining more interest because of its ability to provide absolute values for physical properties of different tissues, such as longitudinal relaxation time (T1), transverse relaxation time (T2) [1]. Several methods have been introduced for quantification of T1, T2 [2–5], but due to the unacceptable scan time, these methods had not been widely used in clinical practice.

Recently, a new method, which is now referred to as multi-dynamic multi-echo (MDME) sequence, enables acquisition of quantitative T1, T2 of the whole brain within approximately 6 min [3]. According to the quantitative data, various contrast images can be created with certain software by manipulating scanning parameters in the single acquisition. It shows promising results in imaging of central nervous system diseases, such as multiple sclerosis (MS) [6], brain tumor [7], Sturge-Weber syndrome [8], bacterial meningitis [9], and stroke [10], as well as diseases in musculoskeletal system [11], spine [12], prostate [13], and breast [14]. In neuroimaging field, brain tissues segmentation can be automatically performed and the volumes of different brain tissues can be calculated [15, 16]. So, it can be potentially used in normal aging and neurodegenerative diseases [17].

Since T1, T2 values represent physical constants that are presumably intrinsic to a given tissue or other material, changing image acquisition parameters theoretically should not alter them; however, model-based derivations of these parameters from real data cannot be expected to be perfectly reproducible. Previous studies showed good accuracy, repeatability, and reproducibility of T1, T2 measurement using different head coils on 1.5T scanner [18] and showed robustness of this sequence across different vendors of 3.0T scanner [19]. Recently, Kang et al. observed that some brain regions of T1 values are slightly changed according to different slice thickness or interslice gap [20]. Some studies showed that brain tissue and myelin volumetry derived from synthetic MRI were robust with different in-plane resolutions in 1.5 T [1] but differences were found in some brain regions in 3.0 T MRI scanner [21]. In clinical multicenter study, we may use MDME sequences with different scan parameters, or change scan parameters to achieve shorter scan time for patients who have difficulty cooperating with the examination. For further expansion of clinical applications of the MDME sequence, the effect of scan parameters, such as echo train length (ETL), acceleration factor and matrix, on the measured quantitative values needs to be investigated.

Hence, the aim of this study was to investigate the effect of scan parameters on T1, T2 relaxation times measured with MDME sequence using gray matter, white matter and cerebrospinal fluid simulation phantoms on 3 T scanner.

## Methods

### MRI acquisition

MRI examinations were performed on a 3 T scanner (SIGNA Pioneer; GE Healthcare, Milwaukee, USA) using a 32-channel head coil. Quantitative MRI was performed using MAGiC (MAGnetic resonance image Compilation) sequence [22]. This sequence is a multisection, multiecho, multisaturation delay method of saturation recovery acquisition that uses a fast spin-echo readout. A single basic block of this quantification sequence consists of 2 phases. In the first saturation phase, a slice-selective saturation pulse with flip angle θ is performed on slice n, followed by subsequent spoiling the signal (“saturation”). In the second acquisition phase, a slice-selective fast spin-echo acquisition is performed on another slice m (“acquisition”), consisting of multiple echoes which are acquired to measure transverse relaxation time (T2). By shifting between slice m and n, a desired delay time can be set between the saturation and acquisition of each specific slice. The longitudinal relaxation time (T1) after a saturation pulse can be retrieved from multiple scans, by using different delay times. Since the number of scans and delay times can be freely chosen, the dynamic range of T1 can also be set as desired [3]. In this way, two echo times and four delay times were used to quantify longitudinal T1 and transverse T2 relaxation times and eight complex images per slice were produced. To retrieve T1, T2 maps, while accounting for B1 inhomogeneity, a least square fit was performed on the signal intensity (I) of images by minimizing the following Eq. (1):

1 $$I = {\text{A}} \cdot {\text{PD}} \cdot {\text{exp}}\left( { - TE/T2} \right)\frac{{1 - \left\{ {1 - {\text{cos}}\left( {B_{1} \theta } \right)} \right\}{\text{exp}}\left( { - TI/T1} \right) - {\text{cos}}\left( {B_{1} \theta } \right){\text{exp}}\left( { - TR/T1} \right)}}{{1 - {\text{cos}}\left( {B_{1} \alpha } \right){\text{cos}}\left( {B_{1} \theta } \right){\text{exp}}\left( { - TR/T1} \right)}}$$

where α is the applied excitation flip angle (90°) and θ is the saturation flip angle (120°). A is an overall intensity scaling factor that takes into account several elements, including sensitivity of the coil, amplification of the radiofrequency chain, and voxel volume [19].

### Phantom study

Gray matter, white matter and cerebrospinal fluid simulation phantoms (named GM, WM, CSF, respectively) produced by Wandong (Beijing Wandong Medical Technology Co., Ltd) were included in the study. GM (WD-TP001) consisted of NiCl_2_·6H_2_O 0.25 g, Agarose 9.6 g, Potassium Sorbate 0.1 g, H_2_O 1000 g; WM (WD-TP002) consisted of NiCl_2_·6H_2_O 0.5 g, Agarose 11 g, Potassium Sorbate 0.1 g, H_2_O 1000 g; CSF (WD-TP003) consisted of CuSO_4_·5H_2_O 0.02 g, H_2_O 1000 g.

**Fig. 1:**
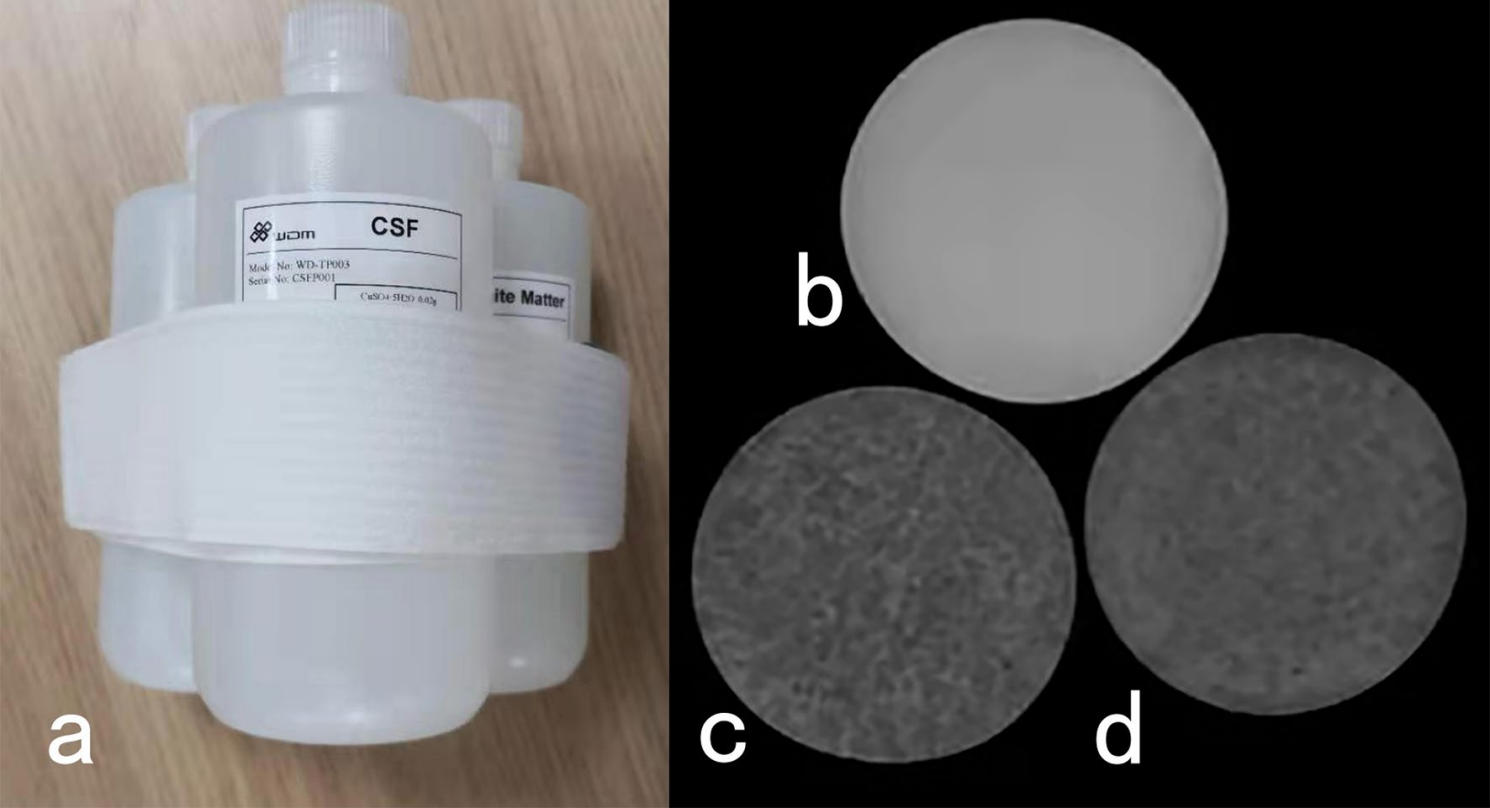
GM, WM, and CSF simulation phantoms (**a**) and Synthetic T2-weighted image of CSF (**b**), WM (**c**) and GM (**d**), acquired from MDME sequence with parameters of group ETL1

Three phantoms were placed together and scanned using MDME sequences with different scan parameters for quantitative MRI (Fig. 1). In order to observe the effect of scan parameters on the measured T1, T2 values, we changed the ETL, matrix, and acceleration factor in a certain range, while trying to keep the other scan parameters unchanged. We selected 4 different ETLs (group ETL1–ETL4), 4 different matrixes (group matrix 1–matrix 4), and 4 different acceleration factors (group phase 1–phase 4). Totally, 12 sets of different scan parameters of MDME sequence were set. TRs (repetition time) and TEs (echo time) would be slightly automatically adjusted with the variations of matrix. The slice thickness was 4 mm, the interslice gap was 1 mm, and the field of view (FOV) was 240 mm × 240 mm.The other detailed sequence parameters were shown in Tables 1, 2, and 3.

**Table 1:** Scan parameters of MDME sequence with different ETLs

	TE1 (ms)	TE2 (ms)	TR (ms)	ETL	Matrix	Bandwidth (kHz)	Phase	Scanning time
ETL1	18.9	94.4	4000	16	288 × 224	25	3.0	2 min, 24 s
ETL2	18.9	94.4	4000	14	288 × 224	25	3.0	2 min, 56 s
ETL3	18.9	94.4	4000	12	288 × 224	25	3.0	2 min, 56 s
ETL4	18.9	94.4	4000	10	288 × 224	25	3.0	3 min, 28 s

**Table 2:** Scan parameters of MDME sequence with different matrixes

	TE1 (ms)	TE2 (ms)	TR (ms)	ETL	Matrix	Bandwidth (kHz)	Phase	Scanning time
Matrix1	14.6	87.5	4000	16	192 × 128	25	3.0	1 min, 52 s
Matrix2	18.9	94.4	4000	16	288 × 224	25	3.0	2 min, 24 s
Matrix3	20.3	101.7	4013	16	320 × 256	25	3.0	2 min, 57 s
Matrix4	23.0	115.0	4450	16	384 × 288	25	3.0	3 min, 16 s

**Table 3:** Scan parameters of MDME sequence with different acceleration factors

	TE1 (ms)	TE2 (ms)	TR (ms)	ETL	Matrix	Bandwidth (kHz)	Phase	Scanning time
Phase1	18.9	94.4	4000	16	288 × 224	25	3.0	2 min, 24 s
Phase2	18.9	94.4	4000	16	288 × 224	25	2.5	2 min,56 s
Phase3	18.9	94.4	4000	16	288 × 224	25	2.0	3 min,28 s
Phase4	18.9	94.4	4000	16	288 × 224	25	1.0	6 min,40 s

The phantoms were scanned 9 times repeatedly on the same scanner, and each scan cycle lasted 3 days (3 acquisition groups) with 12 sets of different scan parameters of MDME sequence based on the modifications of ETL, matrix and phase, respectively. This arrangement should reduce the impact of long acquisition time on measured quantitative values. The entire experiment lasted nearly 1-month (day 1,4,7,10,13,16,19,21,24 for group ETL; day 2,5,8,11,14,17,20,23,26 for group matrix; day 3,6,9,12,15,18,21,24,27 for group phase). The phantoms were placed in scanner for 30 min before each scan. T1, T2 maps were acquired and processed using SyMRI software (SyntheticMR AB, version 8.0.4, Linköping, Sweden). A circle region of interest (ROI) (18.0 cm^2^) was placed in the center of each phantom on T1, T2 maps using RadiAnt DICOM Viewer software (Version 2021.2) to include as much of the circle as possible while avoiding partial volume effect. ROIs were copied and pasted on the images acquired at different times.

### Statistical analysis

According to different sets of scan parameters, the measured values were divided into 3 groups: ETL, Matrix, and Phase. Mean values and standard deviations (SD) for T1, T2 in each group were determined. Coefficients of variation (CVs) were calculated within each group (intragroup CV) and across different groups (intergroup CV). The intergroup CV was calculated using the average values from each of the groups. Statistical analysis was performed using SPSS software (SPSS for Windows, 23.0.0.0, IBM).

## Results

**Fig. 2:**
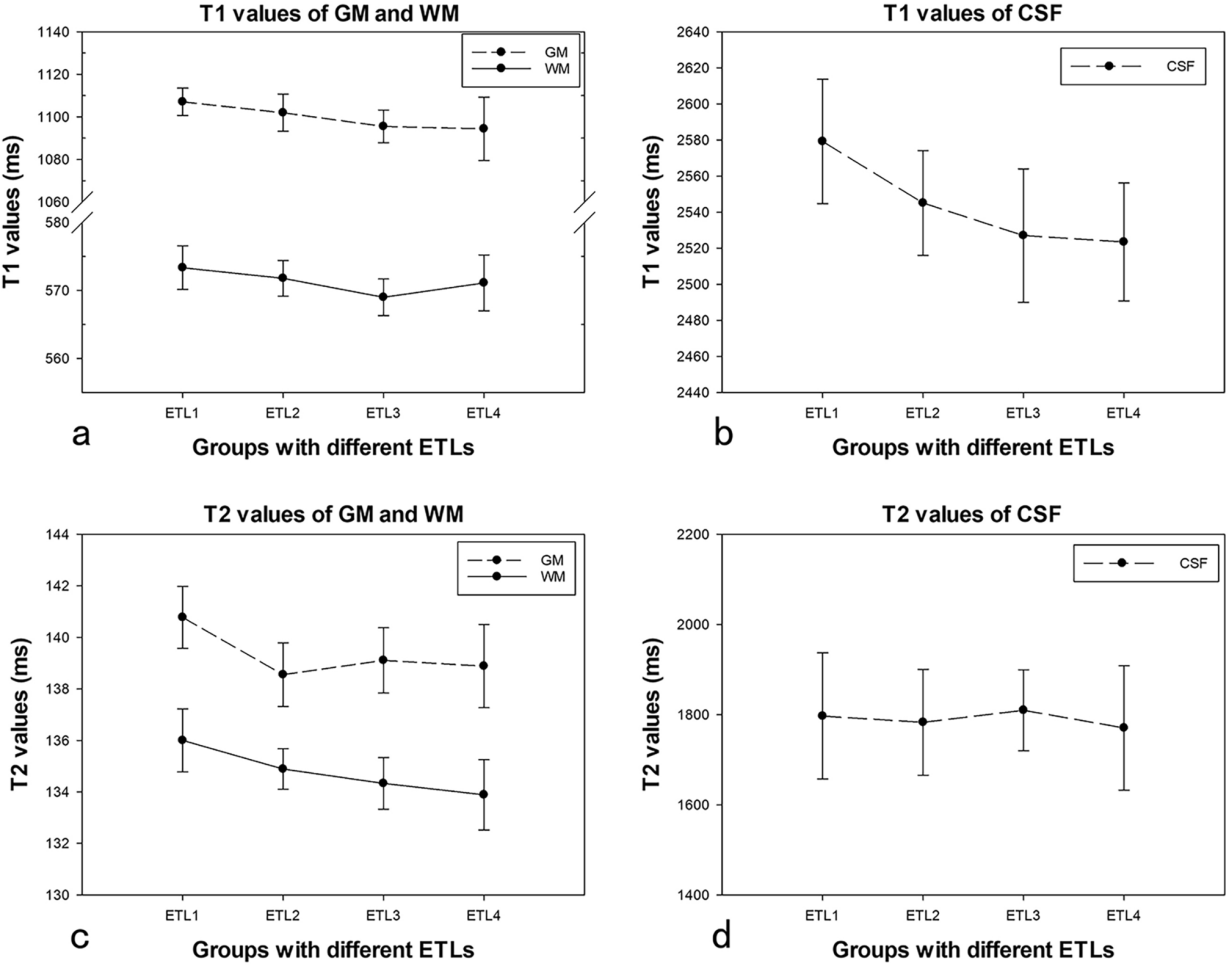
T1 values of GM (**a**), WM (**a**), CSF (**b**), and T2 values of GM (**c**), WM (**c**), CSF (**d**) measured from MDME sequence with different ETLs. The intragroup CVs of all the T1, T2 measurements were less than 2%, except for T2 values of CSF. The intergroup CVs were less than 1%

**Fig. 3:**
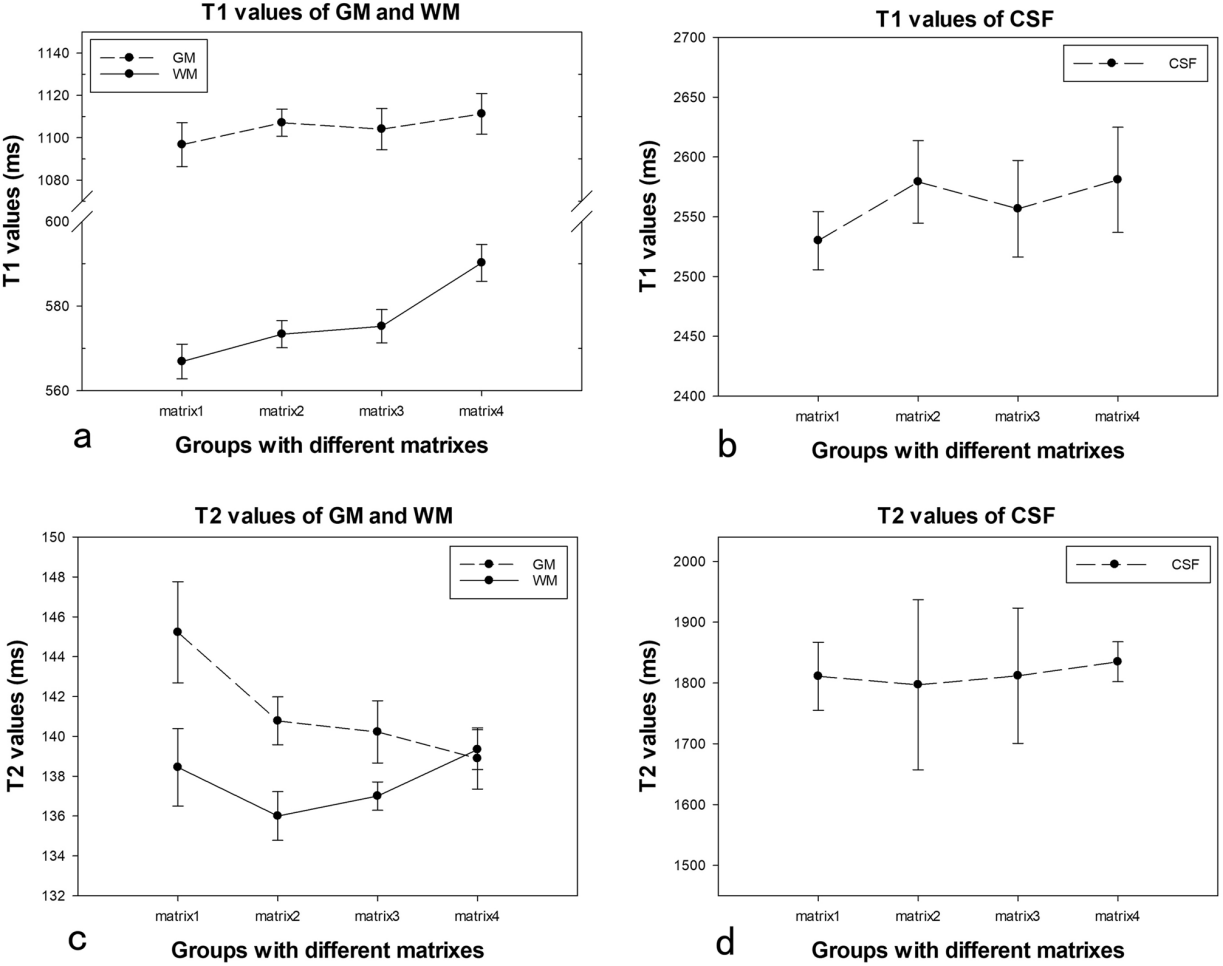
T1 values of GM (**a**), WM (**a**), CSF (**b**), and T2 values of GM (**c**), WM (**c**), CSF (**d**) measured from MDME sequence with different matrixes. The intragroup CVs of all the T1, T2 measurements were less than 2%, except for T2 values of CSF. The intergroup CVs were less than 2%

**Fig. 4:**
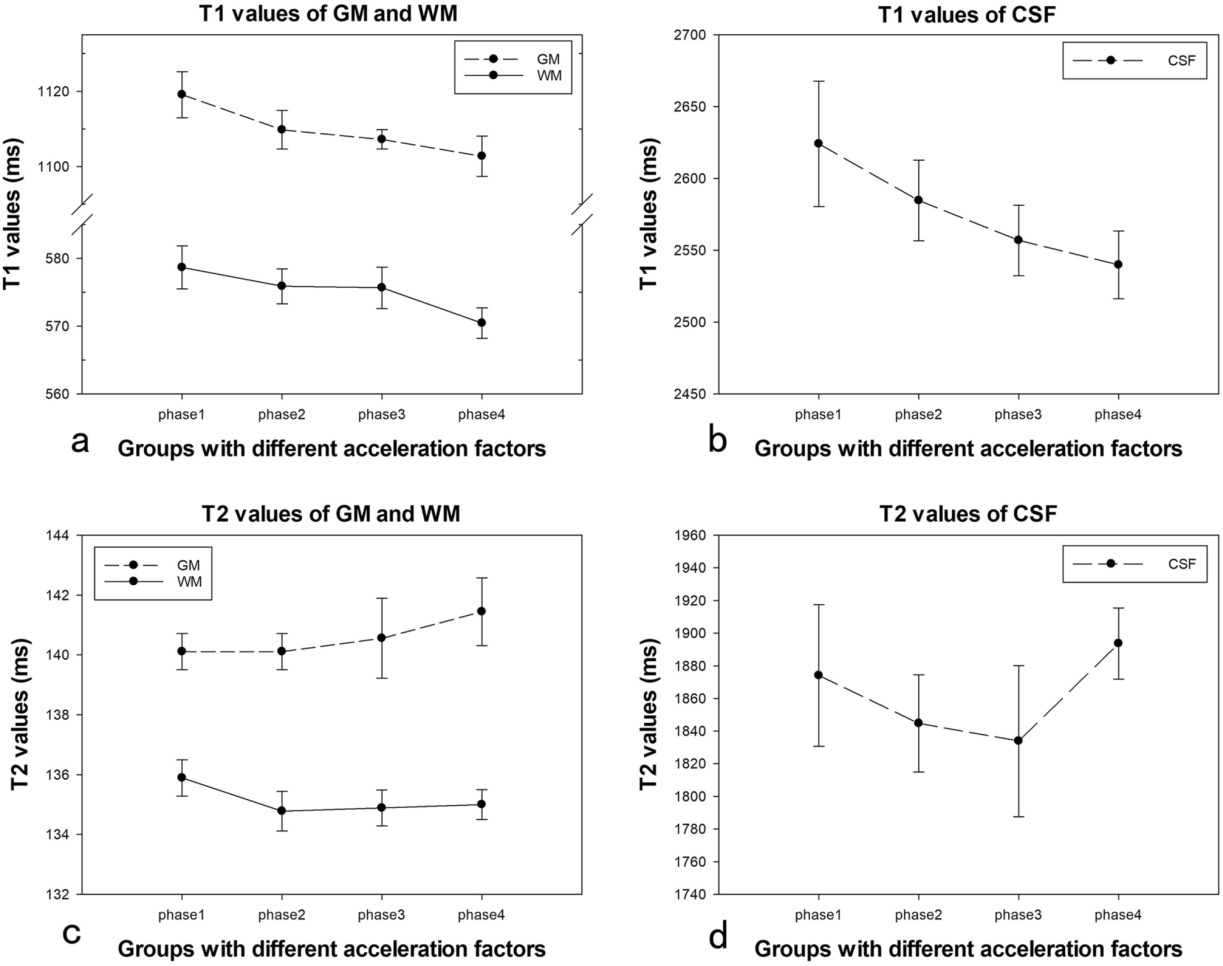
T1 values of GM (**a**), WM (**a**), CSF (**b**), and T2 values of GM (**c**), WM (**c**), CSF (**d**) measured from MDME sequence with different acceleration factors. The intragroup CVs of all the T1, T2 measurements were less than 2%, except for T2 values of CSF. The intergroup CVs were less than 3%

Table 4 showed the mean values and SD of all the T1, T2 measurements on three phantoms in each group. Tables 5, 6, and 7 showed the intragroup and intergroup CVs of T1, T2 measurements on GM, WM, and CSF acquired using MDME sequence with different ETLs (Table 5), matrixes (Table 6), and acceleration factors (Table 7). The intragroup CVs of all the T1, T2 measurements were less than 3%, except for T2 values of CSF. The intergroup CVs of all the quantitative T1, T2 values were less than 3%. For different ETLs, the highest intergroup CV was 1.00% for T1 and 0.80% for T2 (Table 5; Fig. 2). For different matrixes, the highest intergroup CV was 1.70% for T1 and 1.86% for T2 (Table 6; Fig. 3). For variations of acceleration factors, the highest intergroup CV was 0.83% for T1 and 2.17% for T2 (Table 7; Fig. 4).

**Table 4:** Mean values of all the T1, T2 measurements on 3 phantoms with different groups

	T1 (ms)			T2 (ms)		
	GM	WM	CSF	GM	WM	CSF
ETL(1–4)	1100 ± 10.9	571 ± 3.5	2543 ± 39	139 ± 1.6	135 ± 1.3	1790 ± 118
Matrix(1–4)	1104 ± 10.3	576 ± 9.4	2561 ± 40	141 ± 2.9	138 ± 1.8	1813 ± 92
Phase(1–4)	1106 ± 5.5	574 ± 3.5	2565 ± 32	141 ± 1.2	135 ± 0.9	1842 ± 81

**Table 5:** The intragroup and intergroup CVs of T1, T2 values on 3 phantoms measured from MDME sequence with different ETLs

	T1 CVs (%)			T2 CVs (%)		
	GM	WM	CSF	GM	WM	CSF
ETL1-intragroup	0.58	0.56	1.33	0.85	0.89	7.79
ETL2-intragroup	0.79	0.46	1.14	0.88	0.58	6.56
ETL3-intragroup	0.70	0.47	1.46	0.91	0.75	4.98
ETL4-intragroup	1.36	0.72	1.30	1.17	1.02	7.79
Intergroup	0.54	0.29	1.00	0.62	0.62	0.80

**Table 6:** The intragroup and intergroup CVs of T1, T2 values on 3 phantoms measured from MDME sequence with different matrixes

	T1 CVs (%)			T2 CVs (%)		
	GM	WM	CSF	GM	WM	CSF
Matrix1-intragroup	0.95	0.72	0.96	1.75	1.41	3.09
Matrix2-intragroup	0.58	0.56	1.33	0.85	0.89	7.79
Matrix3-intragroup	0.89	0.68	1.58	1.11	0.52	6.13
Matrix4-intragroup	0.86	0.74	1.70	1.11	0.72	1.78
Intergroup	0.53	1.70	0.93	1.86	0.94	0.87

**Table 7:** The intragroup and intergroup CVs of T1, T2 values on 3 phantoms measured from MDME sequence with different acceleration factors

	T1 CVs (%)			T2 CVs (%)		
	GM	WM	CSF	GM	WM	CSF
phase 1-intragroup	0.58	0.56	1.33	0.85	0.89	7.79
phase 2-intragroup	0.46	0.45	1.09	0.43	0.50	1.62
phase 3-intragroup	0.23	0.53	0.96	0.94	0.44	2.52
phase 4-intragroup	0.49	0.39	0.93	0.80	0.53	1.51
Intergroup	0.27	0.50	0.83	0.36	0.37	2.17

## Discussion

The MDME sequence enabled simultaneous acquisition of the physical tissue parameters T1, T2 in an acceptable scan time, and subsequent reconstruction of synthetic images. Promising application of the sequence has been previously reported in neuroimaging [8, 18, 23–25]. Clinical experience is still limited and most relies on the accurate and reproducible study [18, 19].

In this study, we changed the scan parameters of MDME sequence, including ETL, matrix, and acceleration factor within the range of routine clinical use, and evaluated the differences of measured T1, T2 values according to the variation of scan parameters in phantoms using 3T scanner. The observed intergroup CVs were lower than 3%, even lower than the intragroup CVs, indicating that MDME sequence is robust even across different scan parameters in a certain range.

Several studies have investigated the repeatability and reproducibility of MDME sequence. In Hagiwara’s study [19], they measured the quantitative values of the NIST/ISMRM (National Institute of Standards and Technology/International Society for Magnetic Resonance in Medicine) phantom across different scanners and showed that the highest intrascanner CVs was 2.07% for T1 values, 7.60% for T2 values. In our study, the intragroup CVs of T1 values on GM, WM, CSF and T2 values on GM, WM were very low (less than 3%), indicating that the MDME sequence is very reliable in measuring these values. The intragroup CVs of T2 values of CSF showed the highest intragroup CV (7.79%) which was consistent with previous study [19]. This variation is independent of scan parameters and may be related to the MDME sequence itself. The B1 inhomogeneity profiles differ per scan and imperfect gradient refocusing due to eddy currents may decrease signal readout, potentially resulting in an apparently altered T2 relaxation [19]. Another reason may be the limited number of relaxation data points in MDME sequence which makes it less reliable for examining tissues with very short or very long T2 relaxation times. So, it should be cautious to evaluate the T2 measurement of CSF or any other lesions containing fluid using this sequence.

In previous study, Hagiwara et al. [19] demonstrated that the inter-scanner CVs of T1, T2 were 10.86% and 15.27% in phantom data. In our study, the intergroup CVs were less than 3%. In Hagiwara’ study, the inter-scanner CVs of T1, T2 measurements were calculated using data from three different vendors, and in our study, the intergroup CVs of T1, T2 measurements were calculated from the same scanner with different scan parameters. This may be one reason for the bias. On the other hand, the phantoms we used were different from Hagiwara’s and the ROI sizes in our study were much larger, which might result in lower intergroup CVs. In our study, the intergroup CVs of T2 values for CSF were much lower than the intragroup CVs. This suggested that changing scan parameters of MDME sequence, such as ETL, matrix, and acceleration factors, has no obvious effect on the measured quantitative values. This may provide a basis for multicenter study using MDME sequence, and accelerate the acquisition when needed in emergent situations.

There are some limitations in our study. First, we only examine accuracy in T1, T2 values relative to MAGiC sequence variations and not across vendor platforms. The impact of scan parameters on measured quantitative values may be different across different vendors. Second, we only performed phantom measurements but not in vivo measurements. Human brain is much more complex, and the R1 and R2 relaxation might be multiexponential in one voxel under such various microscopic environments. Therefore, the effect of scan parameters on quantitative values of brain should be further investigated.

## Conclusions

In conclusion, our single center study showed that changing the scan parameters of MDME sequence, such as ETL, matrix, and acceleration factor, has no obvious influence (within the difference of 3%) on the measured quantitative T1, T2 values of gray matter, white matter and cerebrospinal fluid simulation phantoms on 3.0T scanner.
